# Lung Ultrasound for diagnosis of acute cardiogenic dyspnea in the Emergency Department – a simeu multicenter study

**DOI:** 10.1186/2036-7902-6-S2-A5

**Published:** 2014-08-27

**Authors:** E Pivetta, M Tizzani, G Porrino, E Ferreri, G Volpicelli, P Balzaretti, A Banderali, A Iacobucci, S Locatelli, F Merletti, I Baldi, G Casoli, E Lupia, GA Cibinel

**Affiliations:** 1 Unita di Epidemiologia dei Tumori e CPO Piemonte, Dipartimento di Scienze Medihe, CeRMS e Universita di Torino, Torino, Italy; 2 Medicina e Chirurgia d'accettazione e d'urgenza, Ospedale "E. Agnelli", Pinerolo, Torino, Italy; 3 Department of Emergency Medicine, Brigham and Women's Hospital, MA, USA; 4 Medicina d'Urgenza, A.O.U. Citta della salute e della Scienza, e Universita di Torino, Torino, Italy; 5 Medicina d'Urgenza, A.O.U. Citta della salute e della Scienza, Torino, Italy; 6 Pronto Soccorso, Ospedale "Cardinal Massaia", Asti, Italy; 7 Medicina d'Urgenza, A.O.U. San Luigi Gonzaga, Orbassano, Torino, Italy; 8 Medicina d'Urgenza, Ospedale "Ordine Mauriziano", Torino, Italy; 9 Medicina d'Urgenza, A.O. Santa Croce e Carle, Cuneo, Italy; 10 Dipartimento di Scienze cardiologiche, toraciche e vascolari, Universita di Padova, Padova, Italy; 11 Medicina d'Urgenza, Ospedale "Martini", Torino, Italy

## Introduction

Acute dyspnea is among the most common presentations to the Emergency Department (ED). Discriminating between cardiac and non-cardiac causes of dyspnea can sometimes be challenging. Lung Ultrasound (LUS), integrated with standard clinical evaluation (i.e. history, physical examination, EKG and arterial blood gas - iLUS) has emerged as a non-invasive bedside valuable technique for diagnosis of various diseases.

## Objectives

Aim of this study was to evaluate diagnostic accuracy of iLUS in identifying cardiac causes of shortness of breath in the ED.

## Patients and methods

This is a multicentric prospective cohort, involving seven Italian EDs (AOU Città della Salute e della Scienza di Torino, “E. Agnelli” General Hospital - Pinerolo, AO Ordine Mauriziano, Turin, Martini Hospital, Turin, AOU San Luigi Gonzaga, Orbassano, Cardinal Massaia Hospital, Asti, and ASO Santa Croce e Carle, Cuneo, all in Piedmont). Neither traumatic nor already invasively ventilated patients presenting to the ED with a principal complaint of shortness of breath were elegible. After initial clinical assessment, emergency physician is asked to indicate the main diagnosis (cardiac or respiratory dyspnea). Then LUS scanning is performed by the same emergency physician and, at this point, a new presumptive diagnosis (iLUS), based on both initial clinical assessment and LUS findings, is recorded. All patients also underwent standard chest radiography (CXR). After discharge, the entire medical records are independently reviewed by two emergency physicians blinded to the LUS results, in order to determine the final diagnosis of patient’s dyspnea (in case of disagreement, a third emergency physician determined final diagnosis).

## Results

1005 patients were enrolled among October 2010 and September 2012. The median age was 77 years (range 18-100 years) and M/F ratio was 1.16. Clinical evalutation had a sensitivity of 85.3% (95% CI 81.8-88.4) and a specificity of 90% (95% CI 87.2-92.4) for the diagnosis of cardiogenic dyspnea. “iLUS” had a sensitivity of 97% (95% CI 95-98.3) and a specificity of 97.4% (95% CI 95.7-98.6). CXR had a sensitivity of 69.5% (95% CI 65.1-73.7) and a specificity of 82.1% (95% CI 78.6-85.2).

## Conclusions

Results of our study showed a high iLUS diagnostic accuracy for the diagnosis of cardiogenic dyspnea among patients admitted to the ED, higher than clinical assessment, and CXR.

